# Peroxisome Proliferator-Activated Receptor δ Agonist, HPP593, Prevents Renal Necrosis under Chronic Ischemia

**DOI:** 10.1371/journal.pone.0064436

**Published:** 2013-05-15

**Authors:** Larisa V. Fedorova, Komal Sodhi, Cara Gatto-Weis, Nitin Puri, Terry D. Hinds, Joseph I. Shapiro, Deepak Malhotra

**Affiliations:** 1 Department of Medicine, The University of Toledo School of Medicine, Toledo, Ohio, United States of America; 2 Department of Physiology and Pharmacology, The University of Toledo School of Medicine, Toledo, Ohio, United States of America; 3 Department of Pathology, The University of Toledo School of Medicine, Toledo, Ohio, United States of America; Max Delbrueck Center for Molecular Medicine, Germany

## Abstract

The Goldblatt’s 2 kidney 1 clip (2K1C) rat animal model of renovascular hypertension is characterized by ischemic nephropathy of the clipped kidney. 2K1C rats were treated with a specific peroxisome proliferator-activated receptor δ (PPARδ) agonist, HPP593. Clipped kidneys from untreated rats developed tubular and glomerular necrosis and massive interstitial, periglomerular and perivascular fibrosis. HPP593 kidneys did not exhibit any histochemical features of necrosis; fibrotic lesions were present only in perivascular areas. Necrosis in the untreated clipped kidneys was associated with an increased oxidative stress, up regulation and mitochondrial translocation of the pro-death protein BNIP3 specifically in tubules. In the kidneys of HPP593-treated rats oxidative stress was attenuated and BNIP3 protein decreased notably in the mitochondrial fraction when compared to untreated animals. In untreated clipped kidneys, mitochondria were dysfunctional as revealed by perturbations in the levels of MCAD, COXIV, TFAM, and Parkin proteins and AMPK activation, while in HPP593-treated rats these proteins remained at the physiological levels. Nuclear amounts of oxidative stress-responsive proteins, NRF1 and NRF2 were below physiological levels in treated kidneys. Mitochondrial biogenesis and autophagy were inhibited similarly in both treated and untreated 2K1C kidneys as indicated by a decrease in PGC1-α and deficiency of the autophagy-essential proteins LC3-II and ATG5. However, HPP593 treatment resulted in increased accumulation of p62 protein, an autophagic substrate and an enhancer of NRF2 activity. Therefore, inhibition of BNIP3 activation by the preservation of mitochondrial function and control of oxidative stress by PPARδ is the most likely mechanism to account for the prevention of necrotic death in the kidney under conditions of persistent ischemia.

## Introduction

Renal artery stenosis (RAS) is a leading cause of renovascular hypertension and ischemic nephropathy eventually developing to end-stage renal disease. In a commonly used animal model for RAS, the Goldblatt’s 2-kidney-1-clip (2K1C), the effects of ischemia can be examined in the clipped kidney and the effects of hypertension in the collateral kidney [1]. Hypoperfusion of the clipped kidney coupled with systemic activation of the renin-angiotensin system results in progressive atrophy of the kidney in the 2K1C model [2]. The exact mechanisms and signaling pathways which trigger renal cell death in stenotic kidneys remain unclear. However, clinical and animal studies demonstrated a critical involvement of oxidants and hypoxia in the genesis of renal atrophy [3], [4].

Under conditions of oxidative stress and hypoxia, programmed cell death pathways are under control of an atypical BH3-only protein BNIP3 (Bcl2 and adenovirus E1B 19 kDa interacting protein 3) [5], [6], [7], [8], [9]. BNIP3 expression is up-regulated in settings of chronic ischemic injury of the heart, brain, liver and neurons [10], [11], [12], [13], [14]. The activity of BNIP3 is dependent upon cellular pH and redox status [15], [16], [17]. Upon activation, BNIP3 is integrated into the mitochondrial membrane and induces permeabilization of the mitochondria and loss of membranous potential, thus activating the mitochondrial cell death pathways [18], [19], [20], [21], [22]. Mitochondria-anchored BNIP3 can also contribute to mitochondria-quality control by triggering proteolytic degradation of mitochondrial proteins and clearance of damaged mitochondria by activation of autophagy [6], [20], [23], [24], [25], [26], [27].

Therapeutic activation of peroxisome proliferator-activated receptors (PPARs), members of the nuclear receptor superfamily of ligand-activating transcriptional regulators, is widely used for management of metabolic and inflammatory diseases. In the kidney, PPARγ activation has a protective effect in diabetic and non-diabetic chronic renal disease [28], [29]. PPARα activation protects kidney from ischemic injuries and renal fibrosis [30], [31], [32], [33], [34]. While PPARδ is essential for protection of the kidney from ischemic acute renal failure and apoptosis [35], [36], the effect of PPARδ activation on the progress of chronic ischemic nephropathy remains unknown.

We examined in the present study whether prolonged treatment of 2K1C rats with the PPARδ agonist, HPP593, has a renoprotective effect on the clipped kidney. Specifically, we studied the effect of HPP593 on the levels of BNIP3 expression, oxidative stress and activation of nuclear respiratory factor 1 (NRF1) and nuclear factor erythroid-derived 2-related factor 2 (NRF2), the main regulatory factors of the intracellular redox balance. In addition, we analyzed expression of mitochondrial proteins, as well as proteins involved in the control of mitochondrial autophagy – Beclin 1, ATG5, LC3 - and biogenesis–PPARs co-activator PGC-1α. We show that HPP593 treatment is cytoprotective in this rat model of ischemic nephropathy and, as such, therapies that activate PPARδ offer a potential approach for the treatment of this disease.

## Materials and Methods

### Antibodies and Reagents

HPP593, the PPARδ agonist (http://www.ttpharma.com/TherapeuticAreas/MetabolicDisorders/Dyslipidemia/HPP593/tabid/118/Default.aspx), was a gift from Transfech Pharmaceuticals (High Point, NC). The following rabbit polyclonal antibodies were used: 1) from Abcam (Cambridge, MA): anti-8 hydroxyquanosine antibody (8-HOG), anti-BNIP3, anti-Keap1, anti-NRF1, anti-NRF2, anti-VDAC1; 2) from Cell Signaling Technology (Beverly, MA): anti-pAMPK/AMPK, anti-Beclin 1, anti-p62; 3) from Novus Biologicals (Littleton, CO): anti-PGC-1α, anti-ATG5; 4) and anti-TFAM antibody (Biovision, Milpitas, CA), anti-LC3 antibody (LifeSpan BioSciences, Seattle, WA). Mouse monoclonal antibodies were: anti-MCAD antibody (Invitrogen, Grand Island, NY), anti-cytochrome c oxidase complex IV antibody (MitoSciences, Eugene, OR), anti-VEGF (Novus Biologicals), and anti-actin antibody (Sigma-Aldrich, Saint-Louis, MO). For lamin detection goat polyclonal antibody was used (Santa-Cruz Biotechnology, Santa-Cruz, CA). Western blot bands were detected with IRDye secondary antibodies (LI-COR Bioscience, Lincoln, NE). Plasma creatinine levels were measured using Creatinine Assay Kit (Cell Biolabs, San Diego, CA).

### Animal Treatment

This study was carried out in strict accordance with the recommendations in the Guide for the Care and Use of Laboratory Animals of the National Institutes of Health. All animal experiments were approved by the Institutional Animal Care and Use Committee of the University of Toledo (Permit number 106706). Male Sprague-Dawley rats (200–250 g body weight; Charles River Lab., Wilmington, MA, USA) were allowed to acclimatize for 4 to 5 days prior to beginning the study. Animals were anesthetized with pentobarbital, and a U-shaped silver clip with an internal gap of 0.25 mm was placed around the left renal artery. All efforts were made to minimize animal suffering. Three groups of rats were studied: sham operated control, 2K1C treated with vehicle (PBS), and 2K1C treated daily with the HPP593 (5 mg/kg body weight) intraperitoneally for four weeks started next day post-surgery. Prior and during the treatment rats were housed, one per a cage, in a temperature-controlled environment (22–24°C) using a 12 hr light/12 hour dark cycle with standard chow (4% fat mouse/rat diet#7001, Harlan-Teklad, Madison, WI) and water provided *ad libitum*. Blood pressure was measured at days 5 and 15 after surgery using the tail cuff method as previously described [37]. Rats were sacrificed on day 30 after the surgery by CO_2_ narcosis. The blood samples were centrifuged at 1,000 g for 10 min 4°C and the plasma was collected and stored at −80°C.

### Renal Morphology and Immunohistochemistry

Formalin-fixed, paraffin- embedded kidney sections were cut 5 µm thick, deparaffinazed and rehydrated. Slides were stained with H&E and Periodic Acid Shift (PAS) Staining Systems (Sigma). For collagen detection, slides were incubated in saturated picric acid containing 0.1% of both Fast Green FCF and Direct Red (Sigma) for 1 hour in the dark. For immunoperoxidase detection of 8-HOG, renal sections were treated with Proteinase K for 20 min, blocked and probed in 1∶4000 diluted 8-HOG antibody in PBS containing 1.5% horse serum at 37°C. For BNIP3 immunohistochemistry, heat-induced antigen retrieval was performed in acetic acid. The sections were blocked and probed with anti-BNIP3 antibody (1∶50) in PBS containing 1.5% of horse serum. After being washed the sections were processed as recommended by ABC protocol (ABC Universal kit; Vector Laboratories, Burlingame, CA). For TUNEL assay, sections were stained with Apoptag Plus Peroxidase Apoptosis Detection Kit (Chemicon International, Temecula, CA) per the manufacturer’s instruction. Images were captured on a Nikon Eclipse 80i microscope equipped with a Nikon camera head DS-Fi1 (Niko, Tokyo, Japan). For quantitative analysis at least 16 randomly chosen fields (4 from each animal) were digitized. Glomerulli perimeters were measured using *NIS Elements for Basic Research* software (Ver. 3.1, Nikon). Collagen volume and oxidative stress levels were determined using the Image J software (http://rsbweb.nih.gov/ij) as previously described [38].

### Tissue Collection and Protein Extraction

Kidneys were immediately excised and either preserved in 4% formalin or immediately frozen at −80°C until used. For protein extraction tissues were homogenized in liquid N_2_ and immediately transferred to RadioImmunoPrecipitation Assay (RIPA) buffer, containing 50 mM Tris-HCl, pH 7.5, 150 mM NaCl, 1% Nonidet P-40, 5% sodium deoxycholate, 0.1% SDS, and protease (cOmplete, Roche Diagnostics) and phosphatase inhibitors cocktails(Halt™, Thermo Scientific) [39].

### Preparation of Cellular Fractions

Mitochondria were extracted as previously described [40]. Kidney tissues were thinly sliced and homogenized in a porter homogenizer, kept on ice, containing 10 mM Tris-HCl buffer, pH 7.4, 0.25 mol/L sucrose, 1 mM EGTA, 0.1 mM PMSF (4 ml/g wet weight) and protease and phosphatase inhibitors cocktail as described above. The homogenate was centrifuged at 1,000×g for 10 min. The supernatant (mitochondrial and cytosolic fractions) was saved. The pellet was homogenized in homogenization buffer and spun down at 300×g for 10 min. The supernatant discarded and the nuclear fraction was collected after washing and spinning the remaining pellet three times with the same buffer at 2,300×g. The supernatant saved after the first centrifugation was centrifuged at 8,000 g for 10 min. The supernatant (cytosolic fraction) and the pellet (mitochondrial fraction) were washed three times for 15 min each at 15,000×g and 8,000×g respectively. Protein concentration was determined using the Modified Protein Assay (Bio-Rad). Mitochondrial yield was estimated per renal wet mass and per extracted proteins since in a significant portion of kidneys from untreated 2K1C rats contained insoluble fibrous proteins.

### Western Blotting

Equal amounts (10–40 µg) of protein were loaded onto SDS-PAGE followed by transfer to Immobilon-FL membrane (Millipore, Billerica, MA) by semi-dry electroblotting. Membranes were then air-dried, rewetted in methanol and incubated in blocking buffer (LI-COR Bioscience). All primary antibodies were used as recommended by the manufacturer. Blots were analyzed using the Odyssey Infrared Imaging system (LI-COR Bioscience) and the results quantitated using the Image J software (http://rsbweb.nih.gov/ij). The area under the curve (AUC) of the specific signal was corrected for the AUC of the actin loading control. The average value for the samples from control rat was set as 1 and other values were calculated accordingly and the ratios compared [39].

### StaRT-PCR for Analysis of Mitochondrial and Nuclear DNA Content

Mitochondrial DNA (mtDNA) content was quantified by competitive PCR according to the method described in detail by Willey et al [41]. Briefly, DNA from kidneys was extracted using DNeasy Tissue Kit (Qiagen, Germantown, MD). DNA was amplified in an Eppendorff Thermal Cycler for 35 cycles with primers for mitochondrial genes – cytochrome B (F: TAA ACT CCG ACG CAG ACA AA, R: 5′: GGT GAT TGG GCG GAA TG) and COXII (F: 5′ GCC CTT CCC TCC CTA C, R: 5′ GAC GTC TTC GGA TGA GAT TA), and actin (R: 5′GAG CGG ACA CTG GCA AAG, F: 5′ CAA AGA CCC ATA GGC CAT CA) for nuclear genes. Competitive templates (CT) were: for cytochrome B –5′CAA AGA CCC ATA GGC CAT CAA CAG ATG CGG CTT AAC ACC C; for COXII -5′GAC GTC TTC GGA TGA GAT TAG GTT TTA GGT CAT TGG TTG G, and for actin –5′CAA AGA CCC ATA GGC CAT CAA CAG ATG CGG CTT AAC ACC C. 25 µl of PCR reaction contained 20 ng of total DNA, 12.5 µl of PCR master mix (Promega, Madison, WI), 2 µl of each primers and 1 μ of CT mixture containing 10^−15^ moles of mitochondrial templates and 10^−11^ moles of nuclear templates. Reaction products were analyzed on Agilent 2100 Bioanalyser Microfluidic CE Device using DNA 1000 LabChip kit (Agilent Technology, Santa Clara, CA). The native template to competitive template ratio was calculated and the number of mitochondrial and nuclear DNA molecules was estimated.

### Statistical Analysis

All data are presented as mean±S.E.M, and mean±SD for blood pressure measurements. Significance of difference in mean values was determined using one-way analysis of variance followed by the Newman-Keul’s post hoc test. Statistical significance was reported at the *p<0.05 and ^##^, **p<0.001 levels.

## Results

### Physiological and Histological Assessments of the Renal Tissue Damage

HPP593 treatment inhibited atrophy of the clipped kidneys and normalized systolic blood pressure in 2K1C rats (Table 1). Plasma creatinine level was elevated in HPP593-treated rats, however this increase was not statistically significant (*p = 0.09*) when compared with sham-operated controls (Table 1).

**Table 1:** Physiological measurements in the experimental groups of the rats.

	Control	2K1C	2K1C+HPP593
	(n = 8)	(n = 8)	(n = 12)
**Relative weight of clipped kidney (%)**	98.4±3.4	38.2±6.0**	71.5±5.4*^#^
**Systolic blood pressure (mmHg)**	124.1±9.4	167±10.8**	135±2.4**^#^**
**Serum creatinine**	0.41±0.08	0.57±0.12	0.62±0.08

The weight of the right kidney was set as 100% and the relative weight of the left (clipped) kidney was recalculated for each animal sample. (n = 8, *p<0.05 and **p<0.001 vs control, ^#^p<0.05 vs 2K1C).

**Figure 1:**
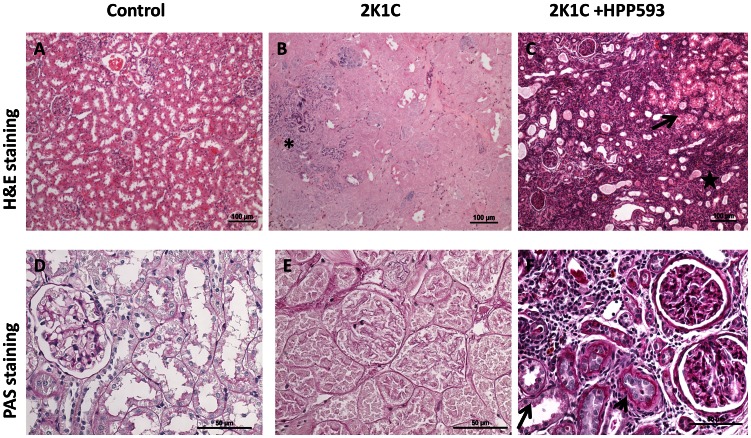
Necrosis of the renal cortex in 2K1C rats and preservation of the renal tissue in HPP593-treated 2K1C rats (H&E and PAS staining). In 2K1C rats, renal architecture is preserved, in some cortical areas where injury has progressed dystrophic calcifications are present (asterisk). Necrotic tubular cells retain their cellular outlines, however their nuclei are lost due to ongoing karyolysis. In the clipped kidneys of HPP593-treated 2K1C rats some renal tubules appear to be intact (arrow). In tubules with dedifferentiated epithelium the lumen is narrowed and basement membranes are multilayered (arrowhead). Other tubules are dilated and filled with proteinaceous cast. Interstitium contains lymphoid infiltrate (star).

**Figure 2:**
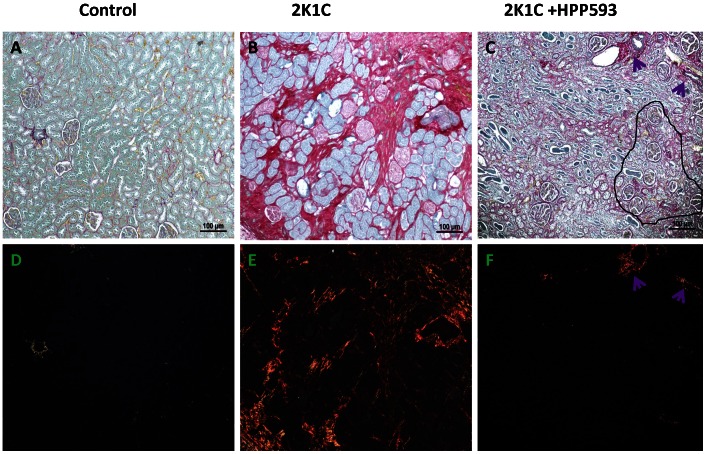
Renal tissue fibrosis in 2K1C and HPP593-treated 2K1C rats (Sirius Red staining). In control kidneys, collagen-specific Sirius Red staining under bright light is observed around glomeruli, basement membrane and blood vessels. Under polarized light, collagen III fibers (yellow-green color) are visible only around blood vessels. In the cortex of 2K1C rats fibrillary collagen I (orange/red color under polarized light) is present in the interstitium and in arteriolar walls. Renal tubules are replaced by the scar tissue in the cortex and at the cortico-medullary junction. Note the sclerotic and crowded glomeruli scattered in the scar tissue of untreated 2k1C rats. In kidneys of HPP593-trearted rats some glomeruli remain crowded (circled in black). Cortical tissue in HPP593-treated 2K1C rats contains large amounts of collagenous matrix as indicated by the intense red color present on these renal sections under bright light. However, unlike in the untreated 2K1C rats, fibrillary collagens I and III are present around blood vessels only (arrows).

**Figure 3:**
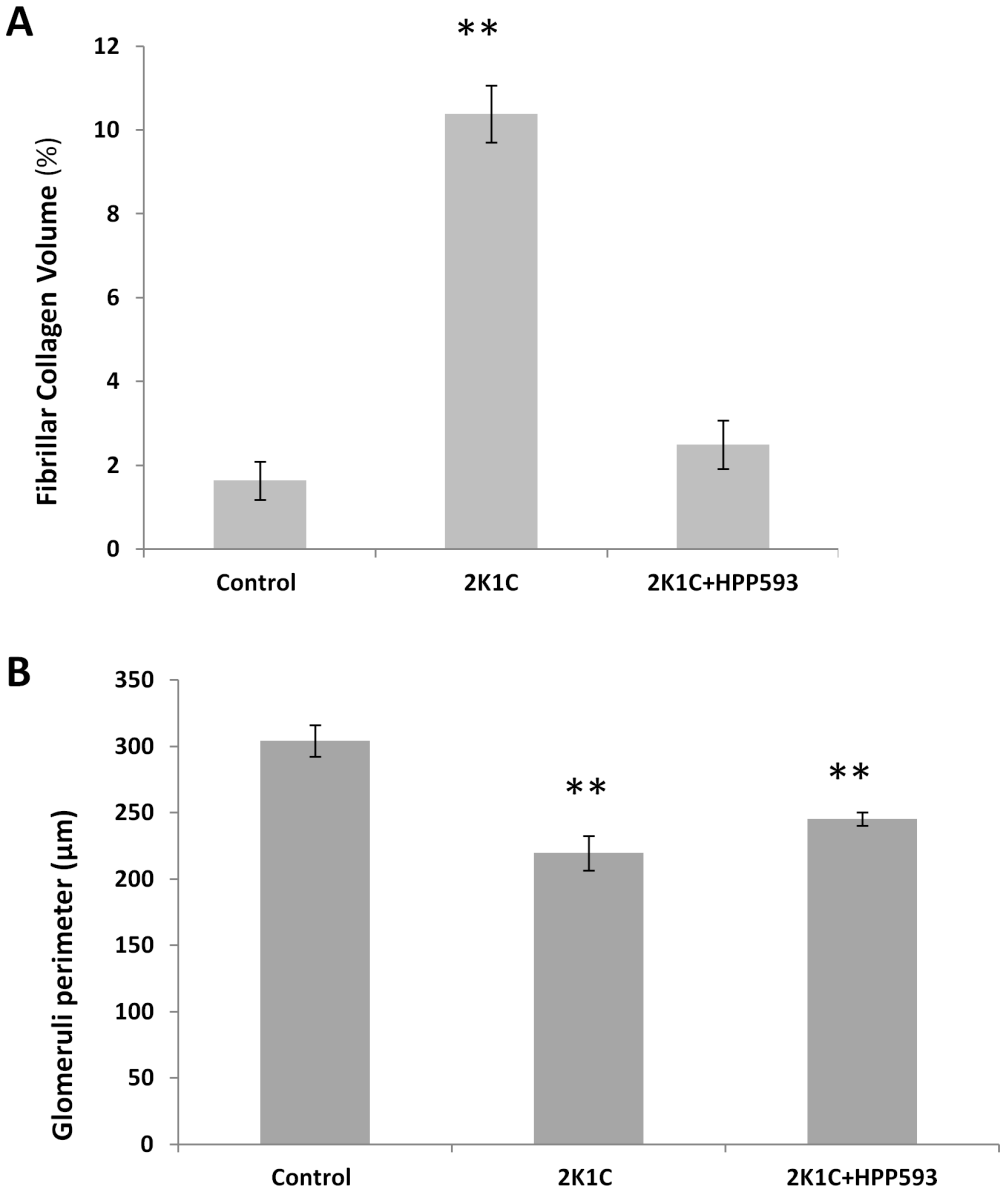
Quantitative analysis of glomerular size (A) and total amounts of fibrous collagens I and III in kidneys of three studied groups. **p<0.001 vs control.

**Figure 4:**
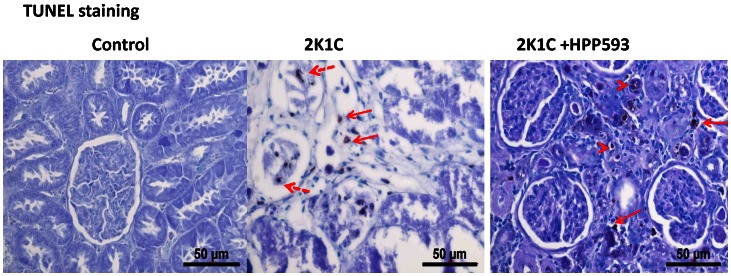
Apoptotic cell death in kidneys of 2K1C and HPP593-treated 2K1C rats (TUNEL staining). Apoptotic cells are absent in the control kidneys. In untreated 2K1C rats, apoptotic cells are present in the cortical scar (arrows with unbroken lines) and in some glomeruli (arrows with broken lines) located close to the cortical capsule. In contrast, in HPP593-treated 2K1C rats apoptotic cells are absent from the glomeruli and present in (arrowheads) or around small atrophic tubules (arrows).

Microscopic examination of clipped kidneys of 2K1C rats showed typical ischemic nephropathy renal injuries [1]. Diffuse coagulative type necrosis was observed in tubular epithelia and in glomeruli in untreated clipped kidneys (Figure 1B,E). In some areas, initial preservation of renal architecture was broken and dystrophic calcifications were present (Figure 1B). Cortical volume was markedly decreased due to replacement of the atrophic tubules by fibrotic tissue (Figure 2B). Globally sclerotic glomeruli surrounded by extracellular matrix, composed mainly of collagen I, were seen in the cortex and at the corticomedullary junction (Figure 2 B, E). Blood vessels were abnormal with thickening of the intima (Figure 2 B, E). In the kidneys of rats treated with HPP593, the renal parenchyma remained viable (Figure 1C,F). Some tubules had an almost normal appearance. Many more tubules were lined with small simplified cells and supported by multilayered basement membrane (Figure 1F) indicating ongoing reparation of the epithelia [42], [43]. At the same time, presence of dilated tubules, tubules with proteinaceous cast in their lumen and crowded glomeruli indicated continuing tubular atrophy (Figure 1C and 2C). Tubular interstitium contained a lymphoid infiltrate (Figure 1 C,F) but was free from fibrosis in HPP593-treated rats (Figure 2 C,F). Elevated fibrillar collagen accumulation was found at perivascular zones only; the levels of total collagens I/III were not significantly higher when compared with sham-operated controls (Figure 3A). The glomerular size was significantly *(p<0.001)* decreased in the clipped kidneys of both untreated and HPP593-treated rats (Figure 3B). HPP593-treatment resulted in thickening of Bowman’s capsule (Figure 1F). In untreated kidneys, apoptotic cells were seen in the sclerotic atubular glomeruli and in the interstitium (Figure 4). In contrast, in HPP593-treated rats, apoptotic cells were found among dedifferentiated tubular epithelia.

### Effect of HPP593 on BNIP3 Activation

**Figure 5:**
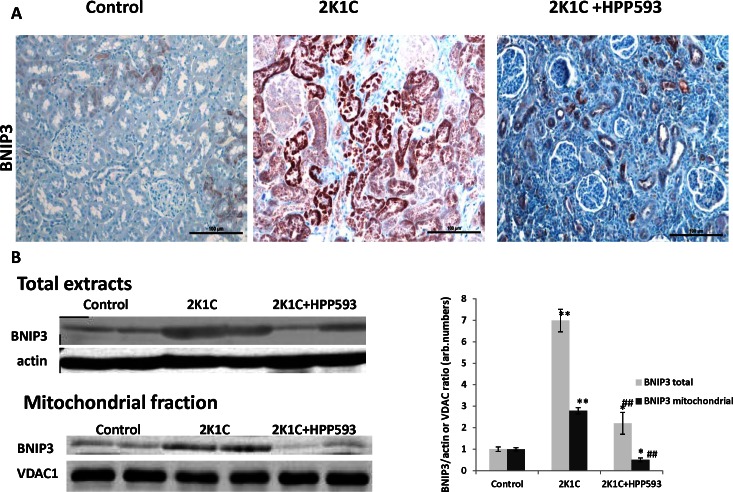
Renal tissue necrosis is associated with an increase in total and mitochondrial content of BNIP3 protein. **A-**immunohistochemical analysis of BNIP3 expression in kidneys. ln control kidneys BNIP3 is expressed in some tubules in the corticomedullary junction. In untreated 2K1C kidneys all necrotic tubules show strong immunoreactivity for BNIP3 protein however there are likely two subpopulations of tubules with different levels of BNIP3 expression. Glomeruli possess a weak immunoreativity for BNIP3. In kidneys of HPP593-treated 2K1C rats BNIP3 positive staining is present in some tubules. **B-** western blotting analysis of BNIP3 expression. Representative immunoblots and densitometry analysis of BNIP3 expression (n = 6) in total and mitochondrial renal extracts and mitochondrial proteins (n = 10) in the mitochondrial fractions. * *p<0.05* and **p<0.001 vs control, *^##^p<0.001* vs 2K1C.

Immunohistochemical analysis with antibody against BNIP3 revealed moderate staining in some tubules located near the corticomedullary junction in control kidneys and in clipped kidneys of HPP593-treated 2K1C rats (Figure 5A). In untreated 2K1C rats all tubules were positive for BNIP3-immunostaining, staining was more intensive when compared to both control and HPP593-treated kidneys. This observation was confirmed by Western blotting analysis (Figure 5B). The total and mitochondrial levels of BNIP3 were substantially increased (7- and 2.5- fold respectively) in the clipped kidneys of 2K1C animals when compared to kidneys from sham operated controls. The total expression of BNIP3 was lower in kidneys of HPP593-treated 2K1C rats when compared to untreated (*p<0.001*), yet levels remained elevated (∼2-fold, *p<0.05*) when compared to controls. However, BNIP3 mitochondrial fraction was decreased by 50% in the HPP593-treated clipped kidneys when compared with control kidneys.

### Effect of HPP593 on Oxidative Stress and NRF2/NRF1 Axis in Clipped Kidneys

**Figure 6:**
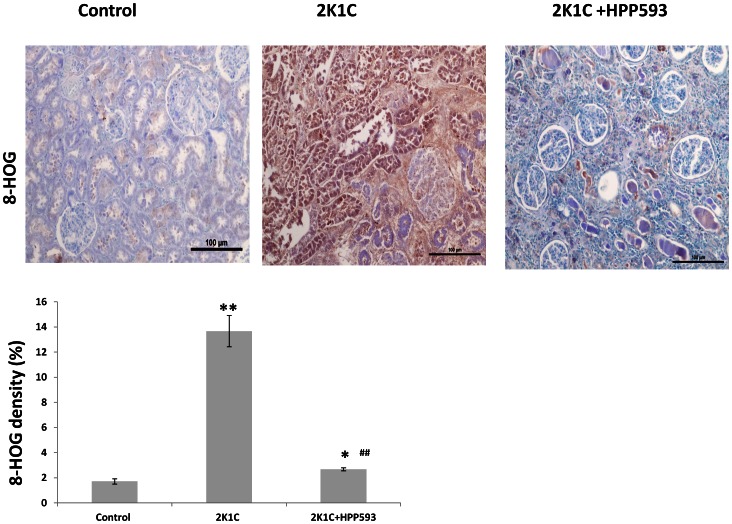
Oxidative stress in kidneys of 2K1C rats. **A-** Immunohistochemical staining of kidney sections with antibody against 8-HOG. **B**- quantitative analysis of 8-HOG staining in the kidneys. * *p<0.05* and **p<0.001 vs control, *^##^p<0.001* vs 2K1C.

**Figure 7:**
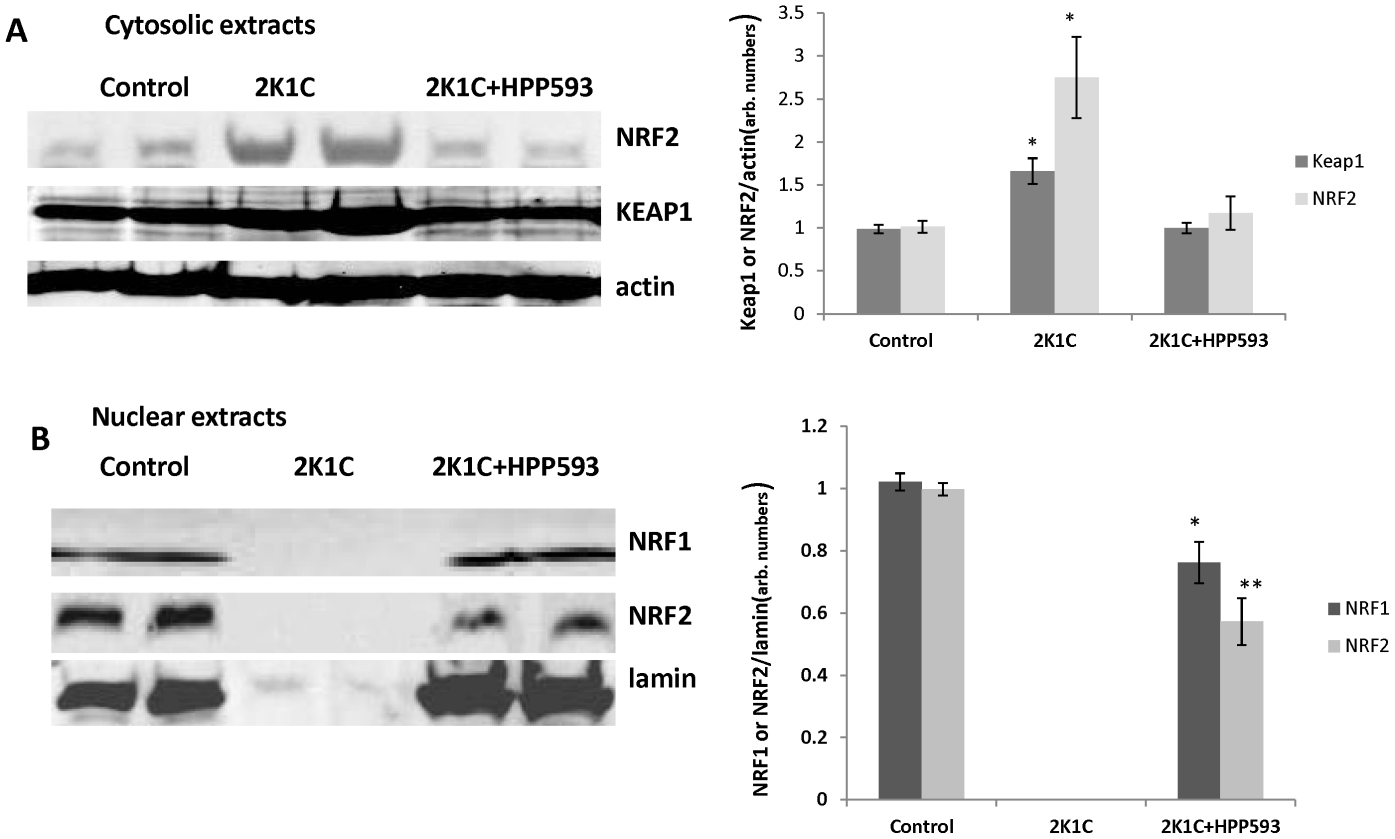
Cytosolic and nuclear levels of the key oxidative defense transcription factors NRF2/NRF1. Representative immunoblots and densitometry analysis of NRF2 and KEAP1 in the cytosolic fraction (A) and NRF1 and NRF2 in the nuclear fraction (B) of renal extracts (n = 6). * *p<0.05* and **p<0.001 vs control.

Up-regulation of BNIP3 protein in clipped kidneys of 2K1C rats was associated with increased oxidative stress as revealed by immunostaining with antibody against 8-HOG and western blotting analysis of KEAP1 (Figures 6, 7). Cytosolic fraction of NRF2, a major oxidative defense transcription factor the degradation of which is regulated by KEAP1 [44], was significantly increased in untreated clipped kidneys when compared to controls *(p<0.05)* (Figure 7A). HPP593 treatment attenuated oxidative damage in the clipped kidneys (Figure 6). Accordingly, there was a significant (*p<0.05*) reduction of KEAP1 levels and a decrease of the cytoplasmic fraction of NRF2 in kidneys of HPP593-treated rats when compared with untreated clipped kidneys (*p<0.05)*. In nuclear extracts from untreated clipped kidneys NRF1 and NRF2 proteins were not detectable due to nuclear dissolusion in necrotic cells (Figure 7B). In HPP593-treated clipped kidneys the levels of NRF1 were ∼20% below that found in control kidneys. Nuclear amounts of NRF2 were decreased by ∼40% in the treated clipped kidneys when compared with sham-operated controls (Figure 7B).

### Effect of PPARδ Activation on Mitochondria

**Figure 8:**
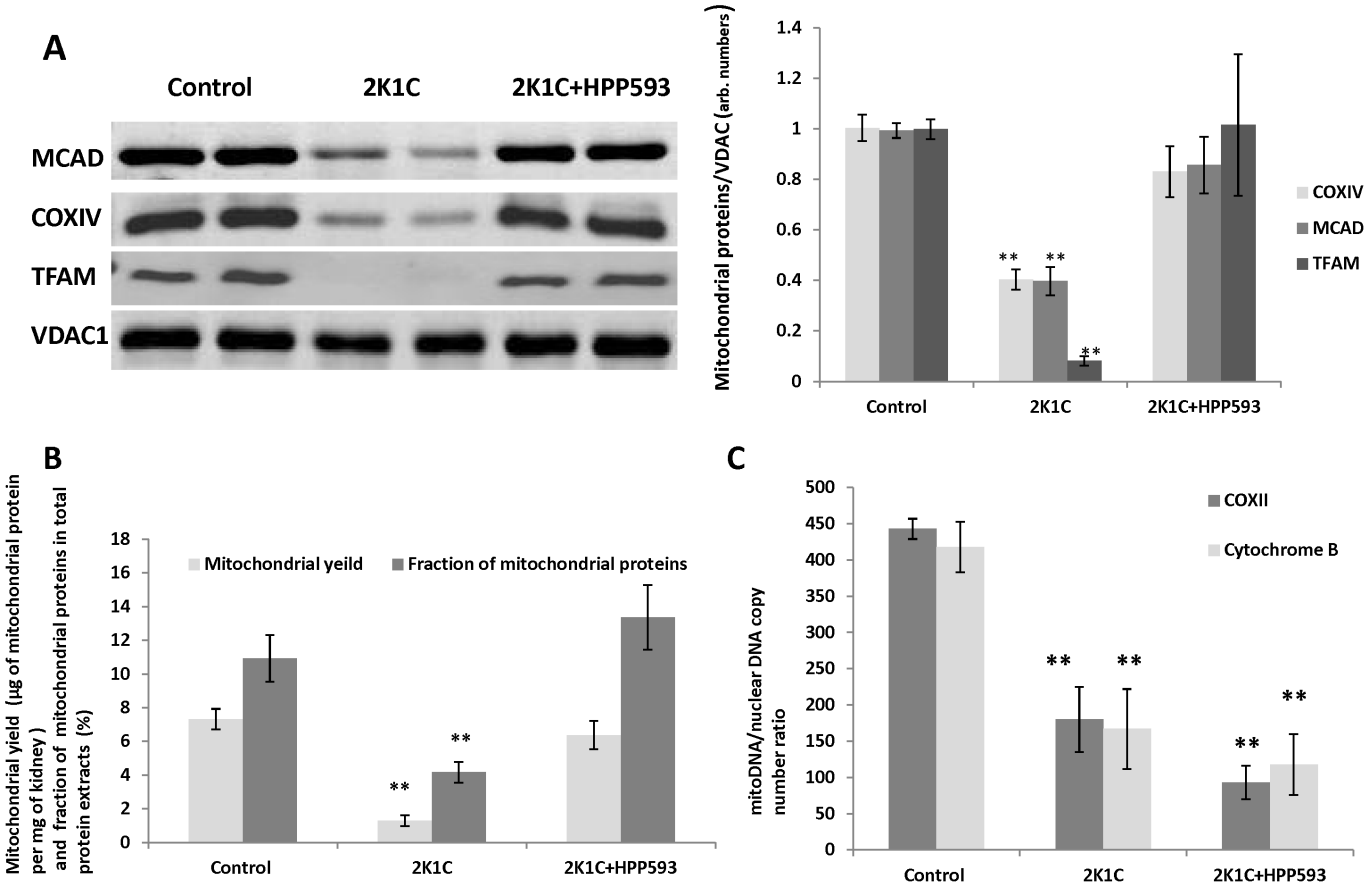
Mitochondrial mass, protein and DNA levels in clipped kidneys of untreated and HPP593-treated 2K1C rats. A- representative western blots and quantitative analysis of mitochondrial protein expression in mitochondrial fractions (n = 16), **p<0.001 vs control and 2K1C+HPP593. **B**-mitochondrial mass estimated as total weight of mitochondria extracted from 1 mg of wet renal tissue (n = 5), **p<0.001 vs control and 2K1C+HPP593. **C**- mitochondrial DNA copy number per nuclear DNA estimated by copy number of mitochondrial genes COXII and Cytochrom B nuclear actin gene (n≥3), **p<0.001 vs control.

We examined the expression of subunit IV of cytochrome c oxidase (COXIV), two mitochondrial matrix proteins, medium chain acyl-CoA dehydrogenase (MCAD) and mitochondrial transcription factor A (TFAM), and outer mitochondrial membrane protein voltage-dependent anion channel 1 (VDAC1). All three proteins, localized to the inner compartment of mitochondria, were down-regulated by more than 50% in the clipped kidney, while VDAC1 levels remained unchanged when compared with control kidneys (Figure 8A). Clipped kidneys from the rats treated with HPP593 retained physiological levels of all three mitochondrial proteins indicating that HPP593 treatment maintains mitochondrial integrity.

The total mitochondrial mass in the clipped kidney of untreated 2K1C animals was decreased by up to 80% when compared to controls (Figure 8B). In the HPP593-treated animals, the total mitochondrial mass in kidney was almost equal to those in sham operated controls. Mitochondrial DNA per nuclear DNA was decreased (up to 80%) in both HPP593-treated and untreated clipped kidneys (Figure 8C). Therefore PPARδ activation preserved physiological levels of mitochondrial mass and proteins in the clipped kidneys in spite of persistent renal ischemia.

### Effect of HPP593 on Markers of Autophagy and Mitochondrial Biogenesis in Clipped Kidneys

**Figure 9:**
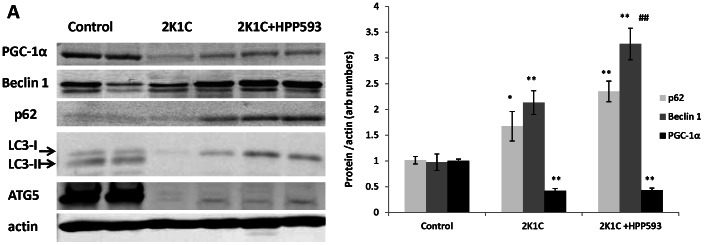
Autophagy impairment and mitochondrial biogenesis in HPP593-treated and untreated clipped kidneys of 2K1C rats. Representative immunoblots and densitometry analysis of the levels of key autophagy proteins and PGC-1α in clipped kidneys of 2K1C rats (n = 6). * *p<0.05* and **p<0.001 vs control, *^##^p<0.001* vs 2K1C.

Western blot analysis revealed that Beclin 1, an essential autophagy inducer [45], was up regulated (Figure 9, *p<0.05*) in both untreated and HPP593-treated clipped kidneys. LC3 protein (the microtubule-associated protein light chain 3) is a marker of the later stages of autophagy [46]. Two forms of LC3 protein (the cytoplasmic LC3-I and membrane-bound, lipid-conjugated LC3-II) were present in control sham operated kidneys indicating physiological levels of basal autophagy. In contrast, both treated and untreated clipped kidneys were deficient of the lipidated LC3-II conjugate. ATG5, which is required for LC3 lipidation [46], [47], was expressed in sham operated kidneys and absent in both treated and untreated clipped kidneys (Figure 9). Autophagy impairment in the clipped kidneys was further confirmed by an increased accumulation of p62 protein in the total extracts from both untreated and HPP593-treated clipped kidneys (two- and three-folds respectively) when compared to sham operated controls, *p<0.001* in both cases (Figure 9). The levels of PGC1-α were reduced by 50% in clipped kidneys (*p<0.01*) and remained unchanged with HPP593 treatment (Figure 9).

### Effect of HPP593 on Mitochondrial Quality and Function and Vasculogenesis in Clipped Kidneys

**Figure 10:**
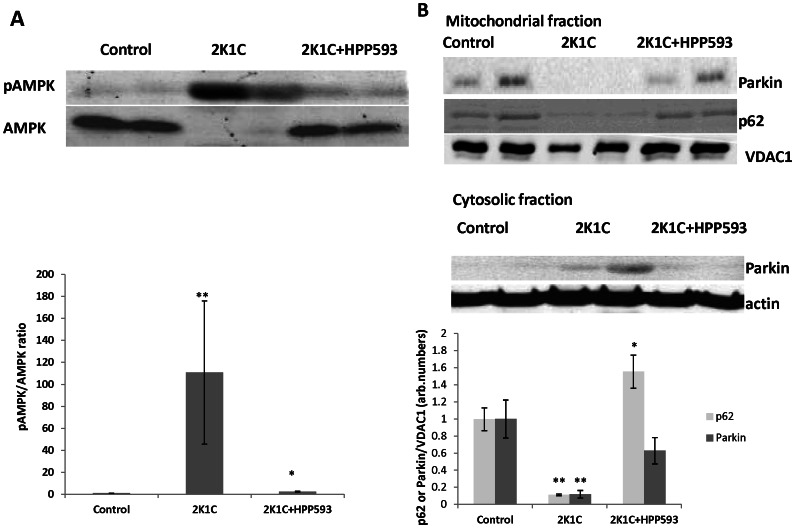
Mitochondrial function in the clipped kidneys of HPP593-treated 2K1C. Representative immunoblots and densitometry analysis of cytoplasmic pAMPK/AMPK expression (A) and p62 and Parkin proteins (B) in cytosolic and mitochondrial fractions (n = 6). *p<0.05 and **p<0.001 vs control.

**Figure 11:**
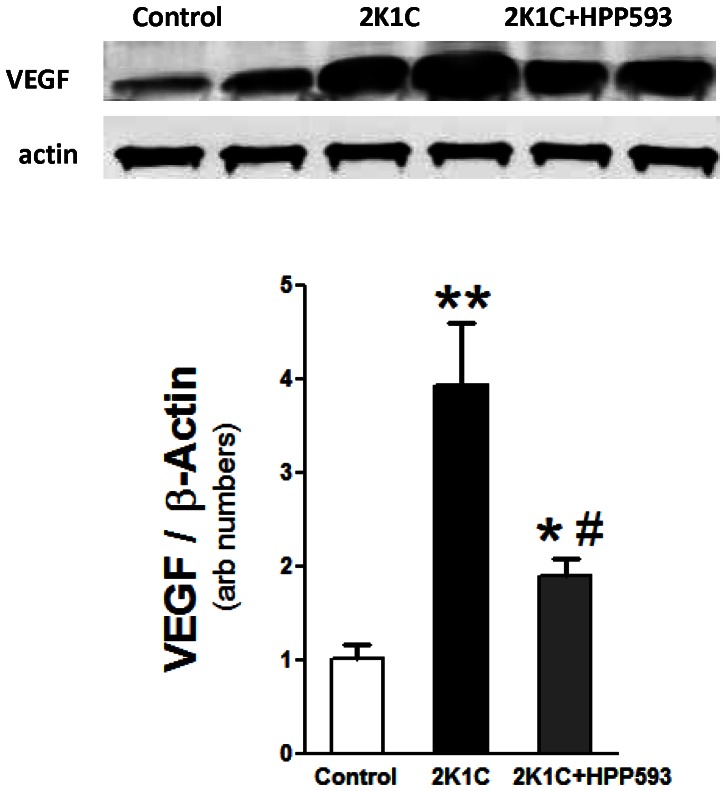
Vascular endothelial growth factor (VEGF) in the clipped kidneys of 2K1C untreated and HPP593 treated rats. Representative immunoblot and densitometry analysis of VEGF expression in kidneys of rats (n = 5). **p<0.01 vs control, *^#^p<0.05* vs 2K1C.

The degradation of mitochondrial proteins in clipped kidneys triggered substantial (*p<0.001)* activation of an energy sensing AMP-activated protein kinase (AMPK) (Figure 10). With HPP593 treatment, the pAMPK/AMPK levels in the clipped kidneys were slightly elevated when compared with those in sham operated controls (*p<0.05*). The levels of Parkin protein, the E3 ubiquitin ligase which binds specifically to depolarized mitochondria [48], were not significantly different in the mitochondrial and cytosolic fractions from the kidneys of control and HPP593-treated animals. In contrast, mitochondrial p62, which is involved in quality control of oxidation-prone proteins [49], was up-regulated (*p<0.05*) in HPP593-treated clipped kidneys. In the untreated clipped kidneys Parkin relocation to mitochondria was inhibited and the mitochondrial portion of p62 protein greatly diminished presumably due to severe damage of mitochondria (Figure 10). VEGF protein was up-regulated in both untreated and treated clipped kidneys (*p<0.01* and *p<0.05* respectively) in response to hypoxia (Figure 11). However, in HPP593-treated 2K1C rats VEGF levels was lower when compared with untreated 2K1C rats.

Thus, preservation of renal mitochondria from bioenergetic collapse and dysfunction rather than increased vasculogenesis appears to be the most likely mechanism of renal protection by HPP-593 under persistent ischemia.

## Discussion

Our data demonstrate that renal atrophy induced by experimental RAS can be inhibited by the pharmacological activation of PPARδ by its agonist, HPP593 (Figure 1, 2). In the kidney, tubular cells are especially vulnerable to ischemia because of high mitochondria volume density necessary for metabolic demand of the ion transport [50]. Three mechanisms can trigger epithelial cell loss depending on the duration and level of hypoperfusion: necrosis, cell detachment and apoptosis [1]. In our experimental setting, tubular necrosis was a primary mechanism involved in renal atrophy in untreated 2K1C rats (Figure 1). Necrotic death of epithelial cells in the clipped kidneys was critically dependent on up-regulation and mitochondrial translocation of the cell death protein BNIP3 since a significant decrease of BNIP3 expression and activation resulted in complete inhibition of necrosis (Figure 1). The exact mechanism(s) by which BNIP3 regulates necrosis is not well understood and is believed to be context–dependent [6], [51], [52]. BNIP3 regulation and activation in renal epithelial cells have never been thoroughly investigated; however studies of cardiac, neuronal, liver and cancerous cells demonstrated that BNIP3 facilitates necrotic cell death via induction of permeabilization of the inner mitochondrial membrane [23]. Under neutral pH, BNIP3 relocation to mitochondria is inhibited and cytosolic BNIP3 is degraded [15], [16]. Progressive hypoxia/ischemia leads to cellular acidosis [53], [54]. In an acidic milieu, BNIP3 is activated upon integration into mitochondrial membrane via its transmembrane domain located in the c-terminus [20], [55]. Oxidative stress triggers oxidation of the BNIP3 n-terminal cysteine residue which in turn promotes BNIP3 homodimerization and further enhances BNIP3 protein stability [17]. In our control kidneys some tubules express BNIP3 presumably as an adaptation to basal levels of hypoxia and oxidative stress present in inner cortex and outer medulla [56], [57]. Studies have demonstrated that BNIP3 has been critically implicated in the pathogenesis of cardiac ischemia [6], [51], [58]. We demonstrated that renal ischemia also is associated with BNIP3 up-regulation and activation. Moreover, BNIP3 pro-death activity can be inhibited even under resisitent hypoxia. Two major mechanisms which may be responsible for BNIP3 deactivation were identified: control of oxidative stress and preservation of mitochondrial function.

Intracellular redox balance is under control of KEAP1/NRF2 system [59]. Under normal conditions steadily expressed NRF2 protein is permanently targeted for ubiquitination and proteosomal degradation by the cytosolic protein KEAP1. Oxidants and electrophiles induce conformational changes of KEAP1 and, thus, stimulate NRF2 release and translocation to the nucleus. NRF2 transcription activity targets broad range of cytoprotective genes including those that are critically involved in antioxidant functions and mitochondrial biogenesis [59], [60]. Oxidative stress was significantly reduced in clipped kidneys of 2K1C rats by HPP593 treatment but remained higher than in sham-operated controls. Elevated oxidant levels in the kidneys of HPP593-treated rats correlated with a decrease in the basal levels of nuclear NRF2. However, HPP593 treatment completely preserved mitochondrial function and proteins suggesting that mitochondria protection might be a primary mechanism of BNIP3 deactivation under hypoxic conditions of stenotic kidney.

In many cell types BNIP3 triggers apoptosis under hypoxia via interaction with the pro-apoptotic Bax/Bak proteins [15], [19], [22]. In untreated clipped kidneys, apoptotic cells were present in the sclerotic glomeruli and intersitium. However BNIP3 immunoreativity was not found in these particular cells (Figure 4, 5). In kidneys of HPP593-treated rats, intensive immunostaining for BNIP3 as well as apoptotic cells were detected in some tubules. Therefore, whether BNIP3 could control apoptotic removal of dedifferentiated epithelial cells and thus facilitate tubular remodeling or atrophy in HPP593-treated kidneys remains to be clarified.

In addition to its pro-cell death activities, BNIP3 facilitates the selective autophagic removal of damaged mitochondria in a variety of cell types [20], [23], [24], [25], [26], [61], [62]. Induction of cell death and autophagy is considered to be two separate and independent functions of the BNIP3 protein. Specifically, inhibition of autophagy can stimulate BNIP3-mediated cell death [20], [24]. Under hypoxia, BNIP3 stimulates autophagy by releasing Beclin 1 from inhibitory interactions with anti-apoptotic BCL-2 proteins [26], [63]. At the later stages of autophagy, the mitochondria-bound BNIP3 can interact with the autophagosome-forming protein LC3-II thus facilitating delivery of dysfunctional mitochondria to the autophagosome [24]. In aging mouse kidneys the BNIP3-induced activation of mitochondrial autophagy under caloric restriction protects tubular cells against hypoxia [64]. However, in our study autophagic machinery was impaired in stenotic kidneys of 2K1C rats as indicated by inhibition of LC3 lipidation and deficiency of ATG5 protein in spite of Beclin 1 up-regulation (Figure 9). Calpain-mediated the N-terminal cleavage of ATG5 was reported in cancer cell lines undergoing apoptosis and in tubular cells in cisplatin nephrotoxicity models [65], [66]. However ATG5 cleavage fragments were undetectable in the present study although antibodies used were raised against N-terminal of ATG5. Notably, mice with genetic ablation of ATG5 gene specifically in proximal tubules develop tubular hypertrophy/degeneration and increased sensitivity to ischemic injury [67], [68].Thus the role and mechanism(s) of ATG5 inhibition in ischemic renal cells remains to be investigated.

Autophagy inhibition leads to p62 accumulation in the cytoplasm (Figure 9) [69], [70]. A multifunctional ubiquitin-binding scaffolding protein p62 contains several functional motifs which allow its interactions with a variety of cell signaling pathways [71], [72]. p62 plays a critical role in cellular adaptation to oxidative stress through its direct binding KEAP1 and following release of NRF2 [70], [73], [74], [75], [76]. In turn, NRF2 positively regulates p62 gene expression independently of oxidative stress [74]. As we showed, p62 levels were increased in untreated clipped kidneys. HPP593 treatment resulted in even higher levels of both total and mitochondrial p62 (Figure 10). Further investigation is needed to clarify the effect of excessive p62 accumulation on the survival of clipped kidneys.

Surprisingly, HPP593 treatment had no effect on mitochondrial DNA content and on the level of PGC-1α, a master regulator of mitochondrial biogenesis and a PPARs co-activator, in the clipped kidneys (Figure 8, 9). However, regulation of mitochondrial DNA copy number is not well understood and is not always correlated with mitochondrial mass [77], [78]. PPARδ stimulates mitochondrial biogenesis through PGC-1α in skeletal muscles and in the heart [79], [80], [81], [82]. However, under conditions of chronic hypoxia in mice, PGC1-α levels and mitochondrial density decrease in the diaphragm but are unchanged in skeletal muscle [83]. Interestingly, this reduction in PGC1-α expression in the diaphragm is associated with an elevation of BNIP3 protein content [83].

The cytoprotective functions of PPARδ, similar to PPARα and PPARγ, are attributed to suppression of oxidative stress and inflammation in a variety of pathological conditions [84], [85]. In particular, PPARδ expression and activation attenuate ischemic damage and reduce the death of cardiac and neuronal cells *in vivo* and *in vitro* [86], [87], [88], [89], [90], [91], [92]. In these studies the cytoprotective role of PPARδ has been linked to suppression of inflammation. Two primary mechanisms, by which activated, agonist-bound PPARδ modulates inflammatory signaling have been found: *i)* deactivation of nuclear factor NF-κB complex via interaction with the p65 subunit [93]; *ii)* release of the transcriptional repressor Bcl6 (B cell lymphoma-6) from its complex with PPARδ [94], [95], [96]. These two mechanisms of PPARδ action, together with PPARδ-dependent up regulation of oxidative stress defense genes, are likely to contribute to attenuation of oxidative stress in kidneys of HPP593-treated 2K1C rats [59], [97]. Interestingly, NF-κB activation can suppress both basal and hypoxia-inducible BNIP3 expression and improve mitochondrial function in ventricular myocytes [98], [99]. These mechanisms of PPARδ actions presumably are cell- and stimulus- specific and might be differently regulated in distinct populations of kidney cells under ischemic condition. Thus, more investigations are needed to clarify mechanisms of PPARδ actions and its interactions with BNIP3, NF-κB and KEAP1/NRF2 systems in the chronically ischemic kidney.

In addition to their anti-inflammatory and antioxidative properties, PPARs are involved in control of angiogenesis. While PPARα and PPARγ activation generally inhibit angiogenesis, different PPARδ agonists may have either pro- or anti-angiogenic effects in various tissues and pathological conditions [100], [101]. VEGF and its receptors are molecular targets of PPARδ agonists [101], [102]. VEGF is constitutively expressed in the kidney and markedly increased in hypoxia [103]. However, overexpression of VEGF was associated with persistent inflammation and kidney damage in various animal models and human conditions [104]. As we found in untreated 2K1C rats VEGF expression was significantly up-regulated yet HPP593 treatment resulted in a decrease of VEGF levels presumably maintaining it within an adaptive tissue protective range.

In summary, we demonstrated that renal atrophy and fibrosis in the 2K1C rat model of ischemic nephropathy can be prevented by the pharmacological activation of PPARδ by its agonist, HPP593. The data presented strongly suggest that: 1) necrotic death of tubular epithelia in clipped kidneys is linked to the up-regulation and stabilization of BNIP3 and to mitochondrial damage; 2) PPARδ activation attenuated oxidative stress and preserved basal mitochondrial function and concomitantly inhibited BNIP3 activation. Significantly, the preservation of renal tissue in HPP593-treated rats was associated with an excessive accumulation of p62 protein due to the impairment of autophagy.
